# Supplementary material for: Renal and hepatic function is preserved following inducible knockout of kynurenine pathway enzymes KMO or QPRT in adult mice
Source: PLoS One. 2025 Dec 4;20(12):e0335906. doi: 10.1371/journal.pone.0335906 (PMC12677463; doi:10.1371/journal.pone.0335906)
Supplement: S1 Table — Abbreviations: KMO, kynurenine 3-monooxygenase; QPRT, quinolinate phosphoribosyltransferase; iKO, inducible knockout; + 7/35D, 7/35 days post tamoxifen; TRP, tryptophan; KYN, kynurenine; KYNA, kynurenic acid; AA, anthranilic acid; 3-HK, 3-hydroxykynurenine; XA, xanthurenic acid; PIC, picolinic acid; QUIN, quinolinic acid. (DOCX) [file pone.0335906.s001.docx]

| **Metabolite** | **Unit** | **Group** | **Concentration**  **(mean ± SD)** | **Range**  **(min-max)** |
| --- | --- | --- | --- | --- |
| TRP | μM | Control (C57BL/6J) | 46.90 ± 16.16 | 40.30 (25.30-65.60) |
|  |  | KMO 1W iKO | 53.47 ± 7.43 | 21.4 (45.60-67.00) |
|  |  | KMO 5W iKO | 53.28 ± 15.44 | 41.70 (30.40-72.10) |
|  |  | QPRT 1W iKO | 62.63 ± 11.76 | 32.40 (48.10-80.50) |
|  |  | QPRT 5W iKO | 54.95 ± 6.87 | 18.00 (43.30-61.30) |
| KYN | μM | Control (C57BL/6J) | 0.74 ± 0.17 | 0.46 (0.56-1.02) |
|  |  | KMO 1W iKO | 1.65 ± 0.59 | 1.39 (1.06-2.45) |
|  |  | KMO 5W iKO | 21.22 ± 12.24 | 30.37 (7.69-38.06) |
|  |  | QPRT 1W iKO | 0.93 ± 0.24 | 0.58 (0.66-1.24) |
|  |  | QPRT 5W iKO | 0.89 ± 0.44 | 1.21 (0.28-1.49) |
| KYNA | nM | Control (C57BL/6J) | 52.60 ± 28.52 | 81.60 (24.60-106.20) |
|  |  | KMO 1W iKO | 131.27 ± 96.42 | 259.10 (31.60-290.7) |
|  |  | KMO 5W iKO | 2362.23 ± 1617.41 | 4508.40 (527.60-5036.00) |
|  |  | QPRT 1W iKO | 28.40 ± 12.28 | 30.30 (13.70-44.00) |
|  |  | QPRT 5W iKO | 36.73 ± 9.71 | 23.60 (26.90-50.50) |
| AA | nM | Control (C57BL/6J) | 42.43 ± 11.39 | 24.40 (32.60-57.00) |
|  |  | KMO 1W iKO | 85.45 ± 31.58 | 78.00 (46.10-124.10) |
|  |  | KMO 5W iKO | 671.55 ± 368.80 | 791.20 (224.70-1015.90) |
|  |  | QPRT 1W iKO | 32.08 ± 28.89 | 64.10 (0.00-64.10) |
|  |  | QPRT 5W iKO | 38.43 ± 12.01 | 30.70 (19.90-50.60) |
| 3-HK | nM | Control (C57BL/6J) | 7.85 ± 9.05 | 21.00 (0.00-21.00) |
|  |  | KMO 1W iKO | 14.35 ± 17.98 | 49.20 (0.00-49.20) |
|  |  | KMO 5W iKO | 9.02 ± 10.26 | 26.20 (0.00-26.20) |
|  |  | QPRT 1W iKO | 4.07 ± 8.16 | 20.40 (0.00-20.40) |
|  |  | QPRT 5W iKO | 8.33 ± 15.78 | 40.10 (0.00-40.10) |
| XA | nM | Control (C57BL/6J) | 39.03 ± 11.92 | 32.70 (23.10-55.80) |
|  |  | KMO 1W iKO | 31.47 ± 21.84 | 52.60 (4.20-54.80) |
|  |  | KMO 5W iKO | 29.74 ± 27.45 | 63.50 (0.90-64.40) |
|  |  | QPRT 1W iKO | 20.17 ± 11.03 | 29.30 (5.20-34.50) |
|  |  | QPRT 5W iKO | 22.60 ± 6.36 | 16.20 (15.10-31.30) |
| PIC | μM | Control (C57BL/6J) | 0.87 ± 0.48 | 1.12 (0.32-1.44) |
|  |  | KMO 1W iKO | 1.86 ± 0.61 | 1.59 (1.33-2.92) |
|  |  | KMO 5W iKO | 1.71 ± 0.59 | 1.61 (1.19-2.79) |
|  |  | QPRT 1W iKO | 1.50 ± 0.78 | 1.76 (0.67-2.43) |
|  |  | QPRT 5W iKO | 1.38 ± 0.33 | 0.88 (1.14-2.92) |
| QUIN | nM | Control (C57BL/6J) | 223.60 ± 77.79 | 197.30 (151.60-348.90) |
|  |  | KMO 1W iKO | 255.30 ± 55.04 | 123.10 (187.60-310.70) |
|  |  | KMO 5W iKO | 330.28 ± 180.67 | 510.20 (99.60-609.80) |
|  |  | QPRT 1W iKO | 402.37 ± 187.56 | 449.50 (146.40-595.90) |
|  |  | QPRT 5W iKO | 1467.63 ± 376.13 | 983.80 (1002.00-1985.80) |
| Serotonin | μM | Control (C57BL/6J) | 1.22 ± 0.92 | 2.35 (0.21-2.56) |
|  |  | KMO 1W iKO | 4.20 ± 4.18 | 11.48 (0.27-11.75) |
|  |  | KMO 5W iKO | 1.22 ± 0.92 | 2.71 (0.22-2.93) |
|  |  | QPRT 1W iKO | 2.49 ± 3.34 | 8.80 (0.01-8.81) |
|  |  | QPRT 5W iKO | 0.16 ± 0.13 | 0.35 (0.01-0.36) |
| KYN*10/TRP | Ratio | Control (C57BL/6J) | 0.17 ± 0.04 | 0.13 (0.12-0.24) |
|  |  | KMO 1W iKO | 0.31 ± 0.10 | 0.27 (0.22-0.48) |
|  |  | KMO 5W iKO | 3.76 ± 1.62 | 4.28 (1.95-6.23) |
|  |  | QPRT 1W iKO | 0.16 ± 0.07 | 0.16 (0.08-0.24) |
|  |  | QPRT 5W iKO | 0.16 ± 0.08 | 0.24 (0.05-0.28) |
| QUIN/KYNA | Ratio | Control (C57BL/6J) | 4.96 ± 1.88 | 5.11 (1.55-6.66) |
|  |  | KMO 1W iKO | 3.38 ± 1.61 | 4.43 (1.52-5.95) |
|  |  | KMO 5W iKO | 0.18 ± 0.11 | 0.31 (0.07-0.38) |
|  |  | QPRT 1W iKO | 14.04 ± 2.58 | 6.82 (10.07-16.89) |
|  |  | QPRT 5W iKO | 43.24 ± 19.40 | 49.51 (24.36-73.88) |
