# Supplementary material for: Renal and hepatic function is preserved following inducible knockout of kynurenine pathway enzymes KMO or QPRT in adult mice
Source: PLoS One. 2025 Dec 4;20(12):e0335906. doi: 10.1371/journal.pone.0335906 (PMC12677463; doi:10.1371/journal.pone.0335906)
Supplement: S2 Table — *p < 0.05, **p < 0.01. (DOCX) [file pone.0335906.s002.docx]

|  | | **Group** | | | | |
| --- | --- | --- | --- | --- | --- | --- |
|  |  | **Control (C57BL/6J)** | **KMO iKO** | | **QPRT iKO** | |
|  |  | **n = 9 (F = 4, M = 5)** | **n = 9 (F = 4, M = 5)** | | **n = 10 (F = 7, M = 3)** | |
| **Clinical chemistry** | | **Value (mean ± SD)** | **Value (mean ± SD)** | **Difference from control** | **Value (mean ± SD)** | **Difference from control** |
| Amylase | U/L | 658.00 ± 89.14 | 660.67 ± 140.17 | NS | 698.40 ± 46.93 | NS |
|  | | | | | | |
| **Hematology** | | **n = 10 (F = 4, M = 6)** | **n = 10 (F = 5, M = 5)** | | **n = 12 (F = 9, M = 3)** | |
| Mean cell volume (MCV) | fL | 44.60 ± 1.17 | 43.7 ± 1.25 | NS | 43.67 ± 1.23 | NS |
| Mean cell hemoglobin (MCH) | pg | 13.02 ± 0.25 | 12.89 ± 0.66 | NS | 13.78 ± 0.75 | ** |
| Mean cell hemoglobin concentration (MCHC) | g/dL | 28.22 ± 3.55 | 29.51 ± 1.75 | NS | 31.66 ± 2.33 | * |
| Red blood cell distribution width, coefficient of variation (RDWc) | % | 19.33 ± 0.52 | 20.6 ± 0.81 | ** | 21.07 ± 1.38 | ** |
| Red blood cell distribution width, standard deviation (RDWs) | fl | 30.40 ± 0.76 | 31.48 ± 1.48 | NS | 30.47 ± 0.79 | NS |
| Mean platelet volume (MPV) | fl | 6.13 ± 0.38 | 6.00 ± 0.39 | NS | 6.20 ± 0.26 | NS |
| Plateletcrit (PCT) | % | 0.22 ± 0.09 | 0.27 ± 0.09 | NS | 0.19 ± 0.09 | NS |
| Platelet distribution width, coefficient of variation (PDWc) | % | 29.82 ± 1.10 | 30.02 ± 1.55 | NS | 30.09 ± 1.71 | NS |
| Platelet distribution width, standard deviation (PDWs) | fl | 7.00 ± 0.56 | 7.15 ± 0.84 | NS | 7.33 ± 1.08 | NS |
