# Supplementary material for: Renal and hepatic function is preserved following inducible knockout of kynurenine pathway enzymes KMO or QPRT in adult mice
Source: PLoS One. 2025 Dec 4;20(12):e0335906. doi: 10.1371/journal.pone.0335906 (PMC12677463; doi:10.1371/journal.pone.0335906)

Computer-generated WES "blot" outputs (uncropped and unadjusted) for KMO and QPRT in liver and kidney tissue

Computer-generated blot images produced by the ProteinSimple WES automated capillary electrophoresis system. These images are not traditional Western blots. Instead, they are software-generated representations created from the underlying electropherogram peak data to provide a format familiar to readers. The raw electropherogram traces were the actual data analyzed in this study (see Supporting Information). The images shown here are uncropped, unadjusted, and presented exactly as exported from the WES software.

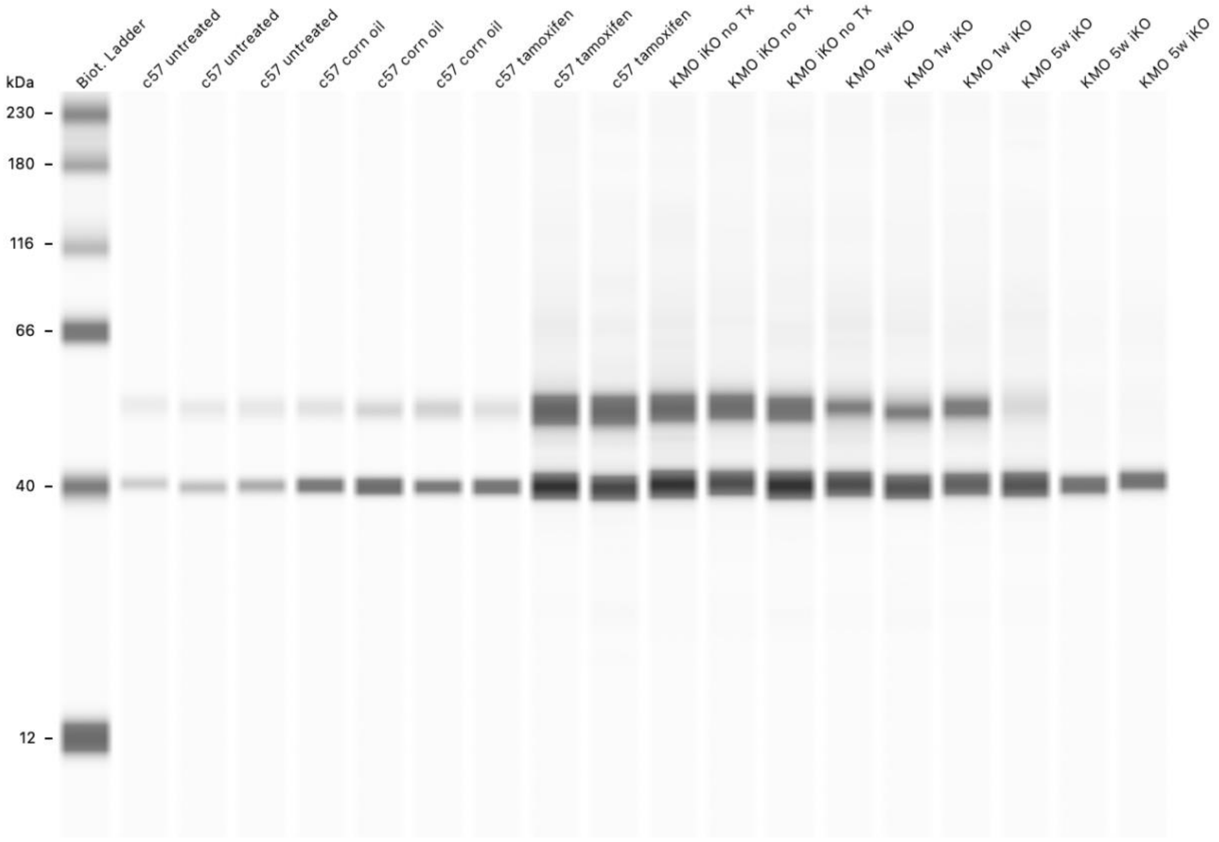

KMO kidney – Uncropped, unadjusted computer-generated WES "blot" output showing KMO (~53 kDa) and GAPDH (~40 kDa) expression in kidney samples (n = 3 per group).

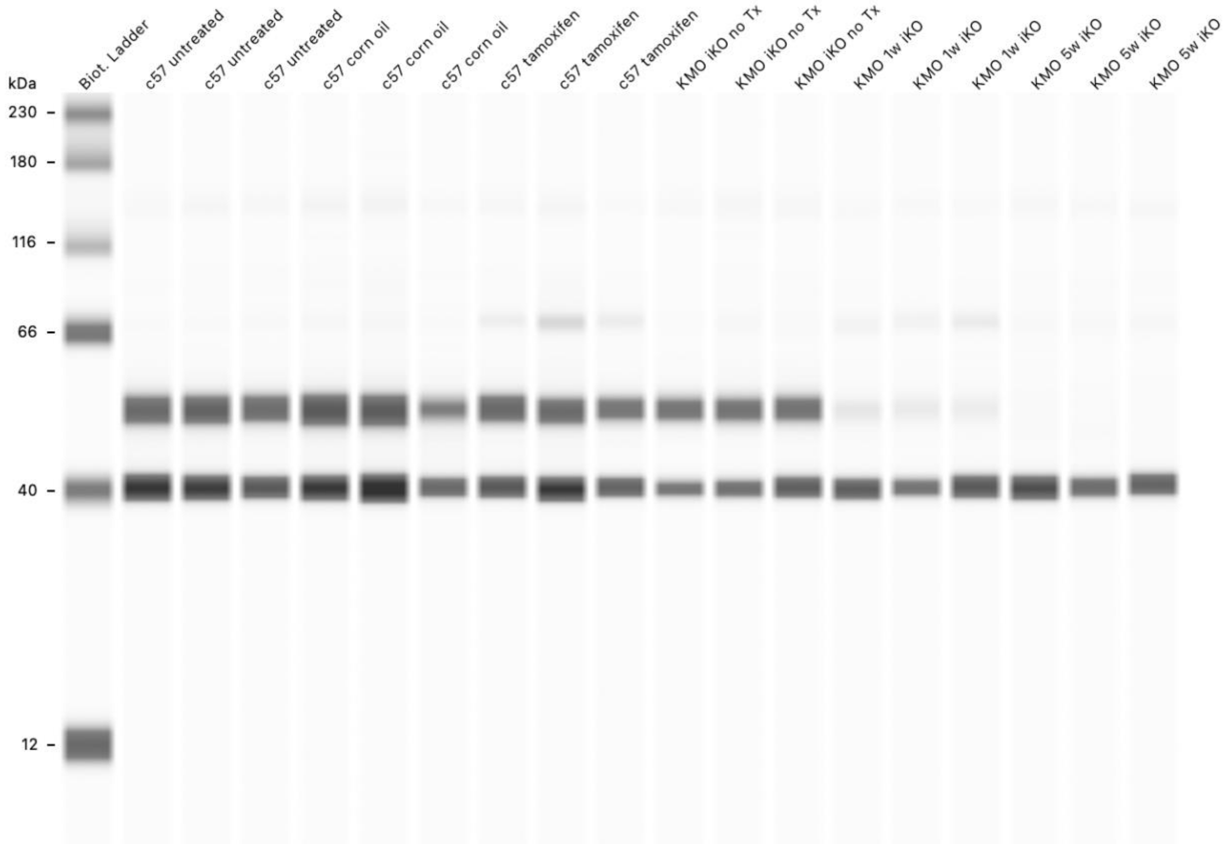

KMO liver – Uncropped, unadjusted computer-generated WES "blot" output showing KMO (~53 kDa) and GAPDH (~40 kDa) expression in liver samples (n = 3 per group).

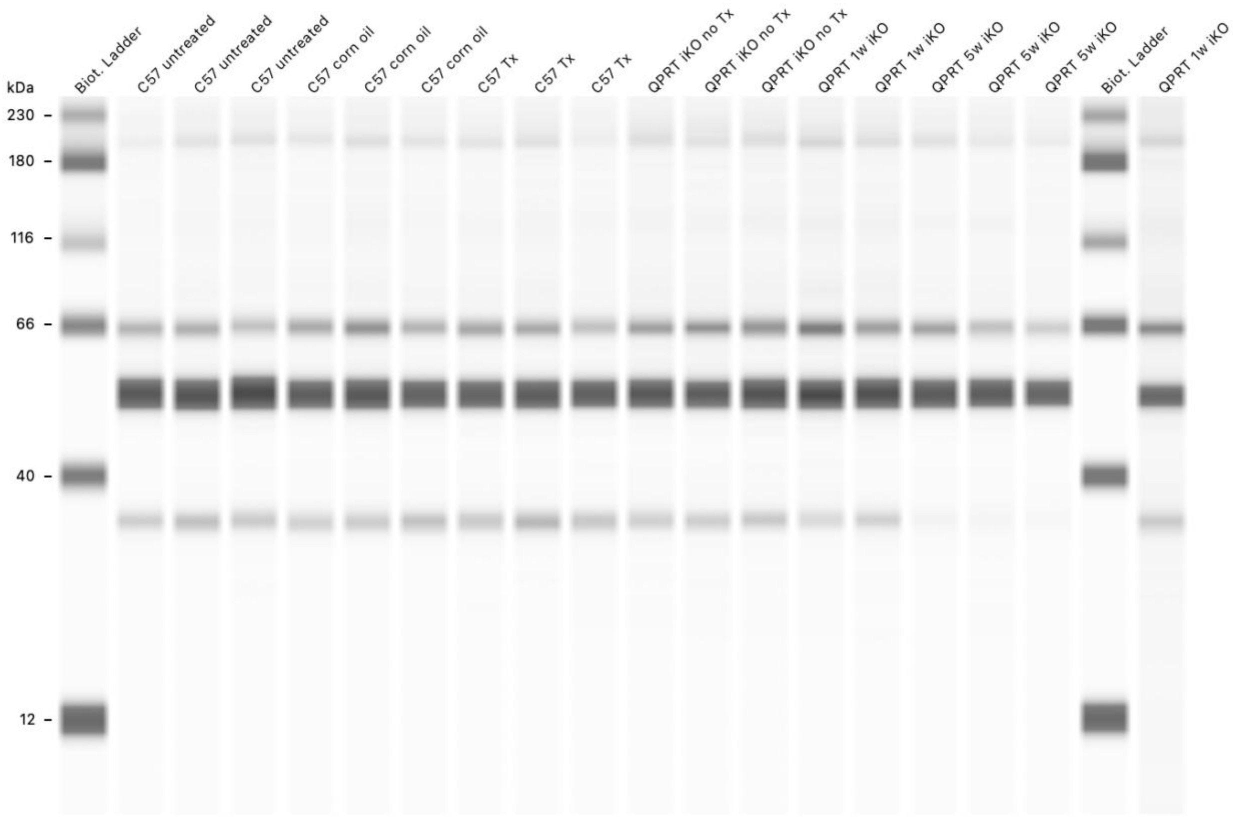

QPRT kidney – Uncropped, unadjusted computer-generated WES “blot” output showing QPRT (~35 kDa) and β-tubulin (~54 kDa) expression in kidney samples (n = 3 per group).

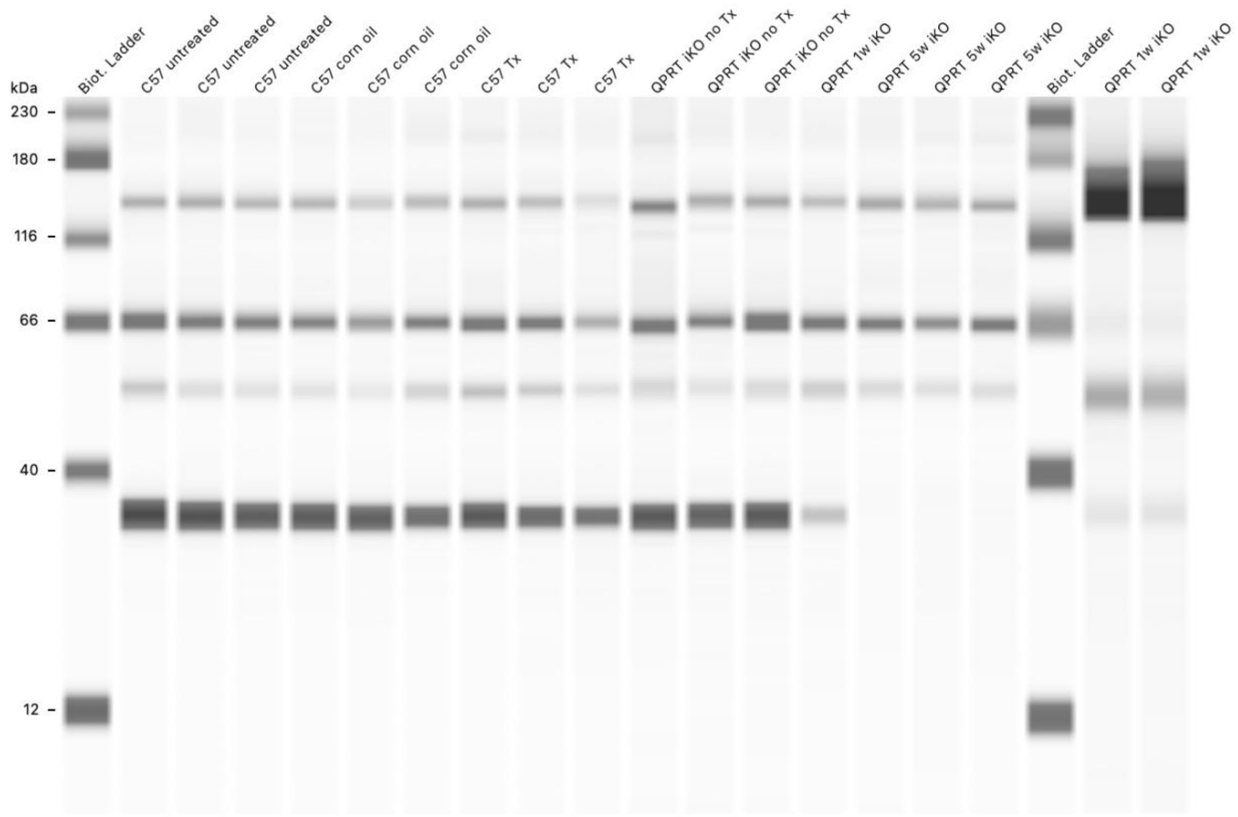

QPRT liver – Uncropped, unadjusted computer-generated WES "blot" output showing QPRT (~35 kDa) and β-tubulin (~54 kDa) expression in liver samples (n = 3 per group).

Simple Protein WES automated capillary western blot raw electropherogram peaks of KMO expression mouse kidney.

Peaks at ~40 kDa and ~53 kDa represent GAPDH and KMO, respectively. Each panel shows a raw electropherogram trace for individual mouse kidney samples (n = 3 per group).

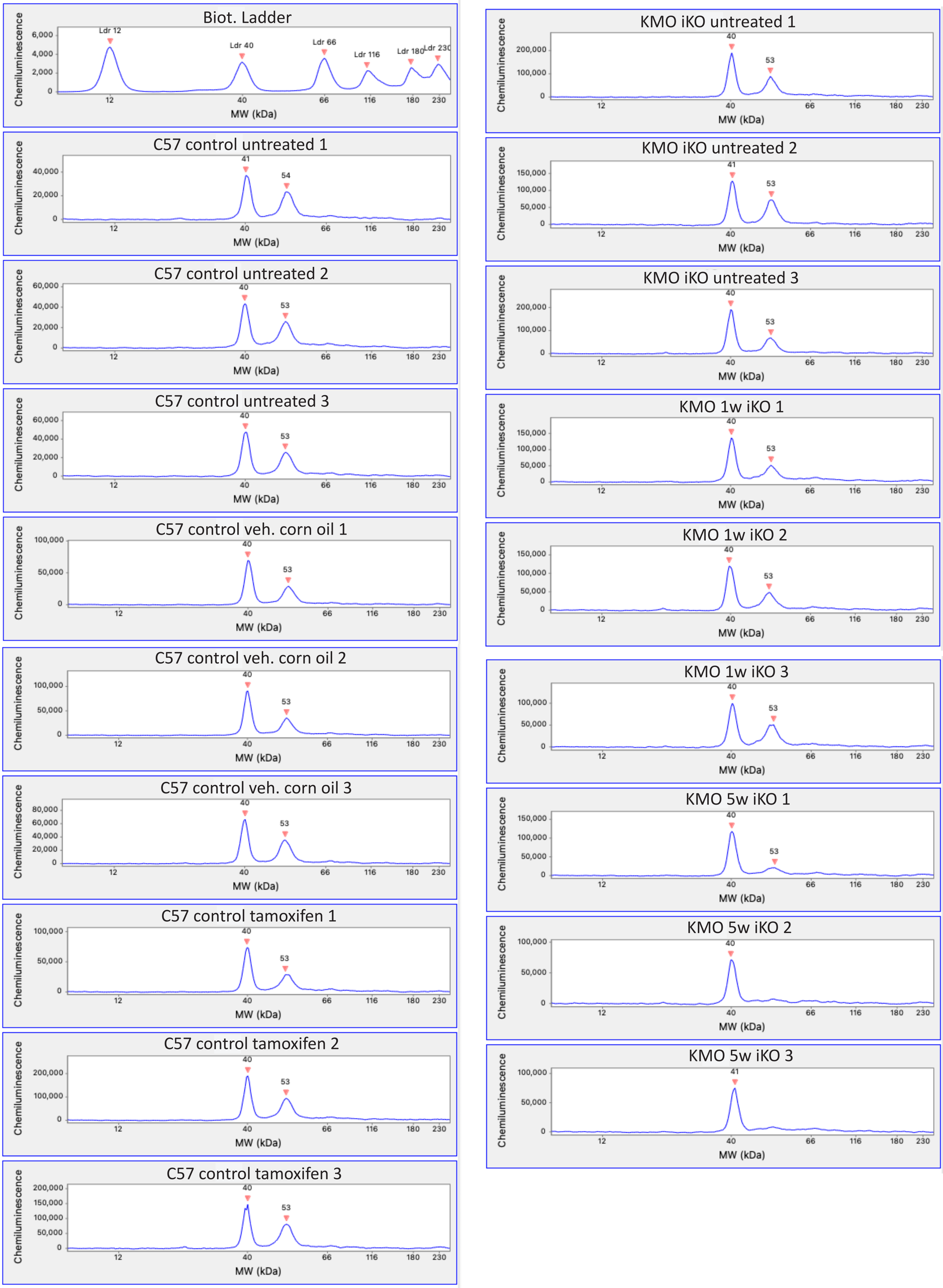

Simple Protein WES automated capillary western blot raw electropherogram peaks of KMO expression mouse liver.

Peaks at ~40 kDa and ~53 kDa represent GAPDH and KMO, respectively. Each panel shows a raw electropherogram trace for individual mouse liver samples (n = 3 per group).

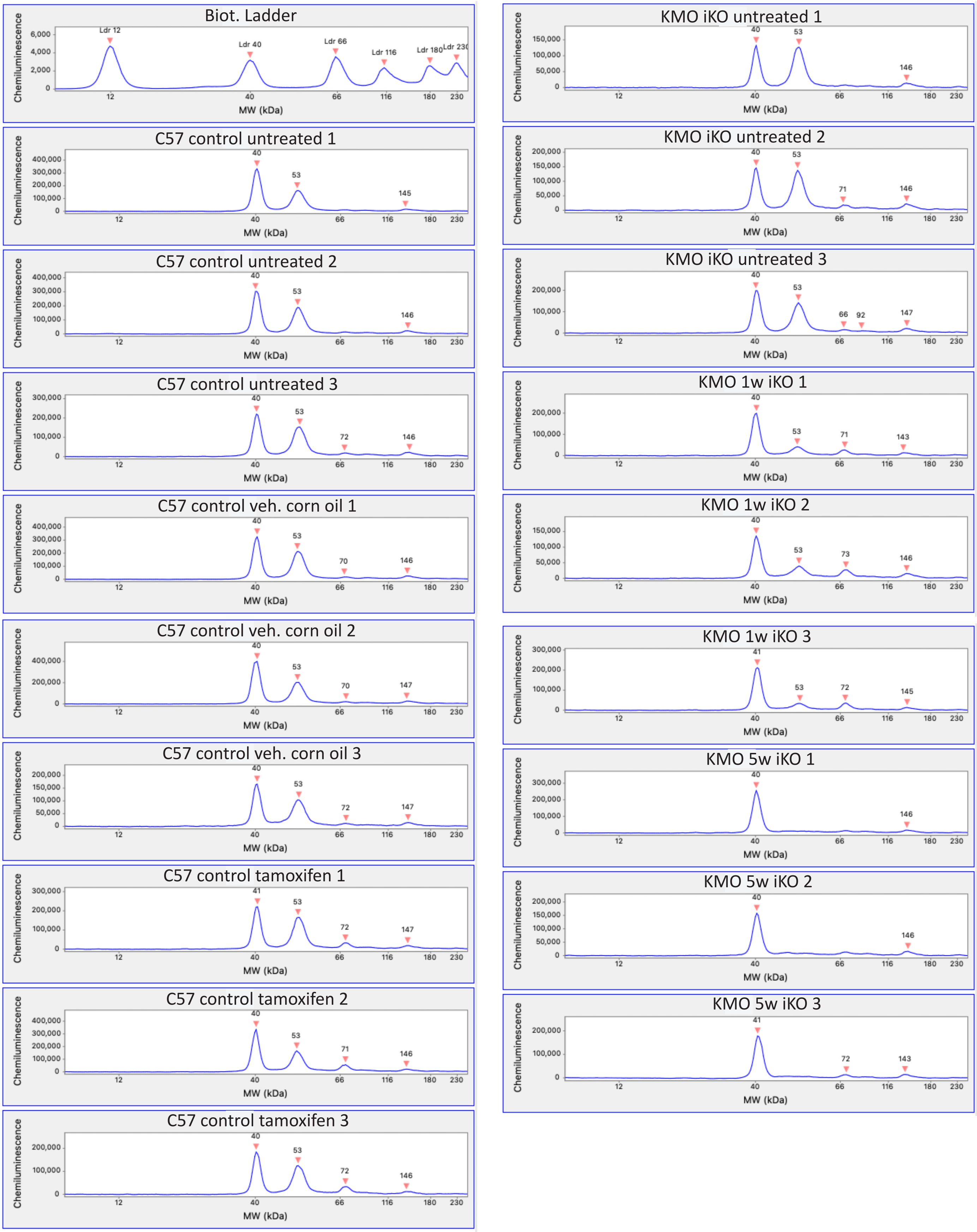

Simple Protein WES automated capillary western blot raw electropherogram peaks of QPRT expression in mouse kidney.

Peaks at ~35 and ~54 kDa represent QPRT and beta tubulin, respectively. Each panel shows a raw electropherogram trace for individual mouse kidney samples (n = 3 per group).

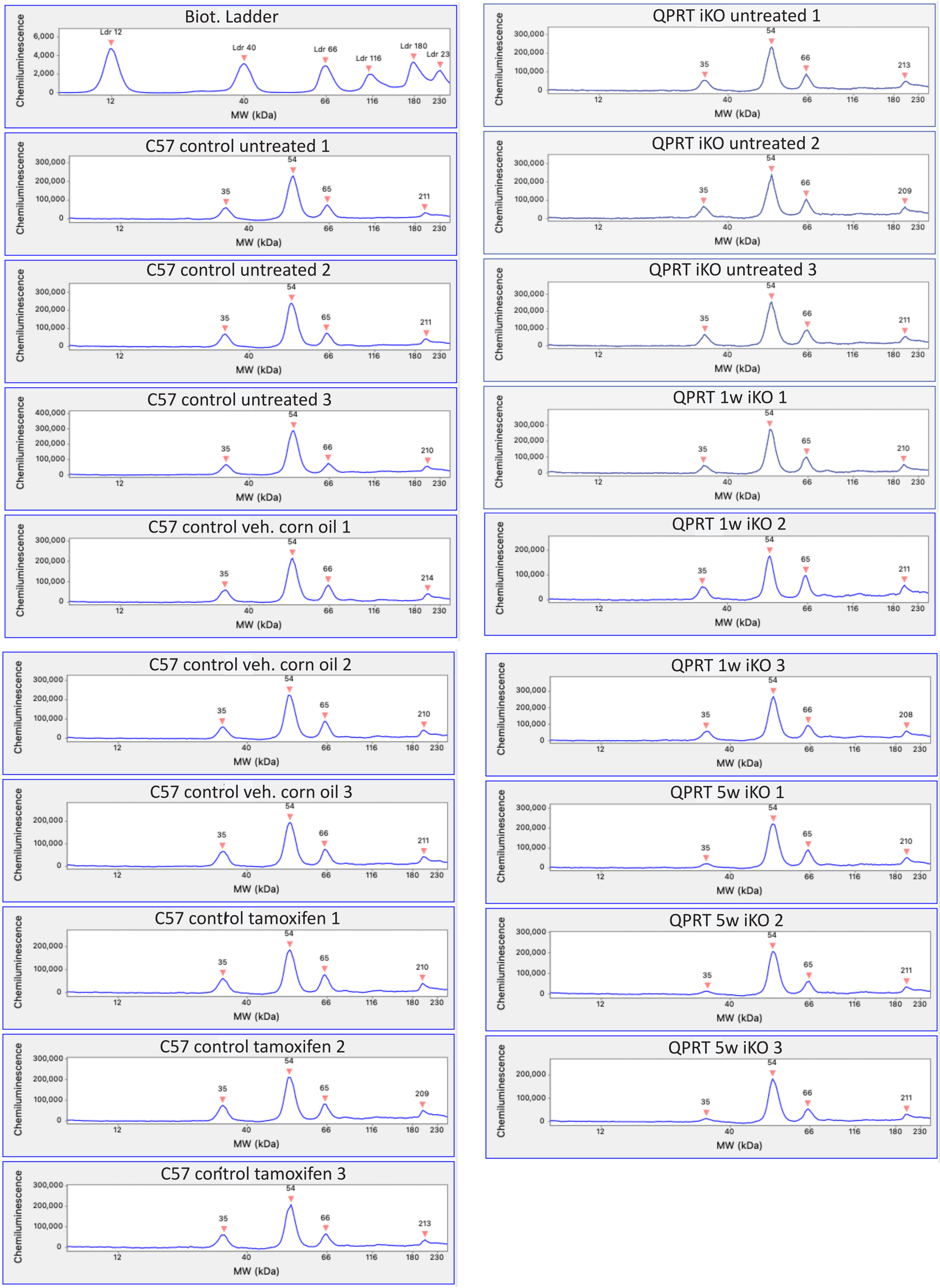

Simple Protein WES automated capillary western blot raw electropherogram peaks of QPRT expression in mouse liver.

Peaks at ~35 and ~54 kDa represent QPRT and beta tubulin, respectively. Each panel shows a raw electropherogram trace for individual mouse liver samples (n = 3 per group).

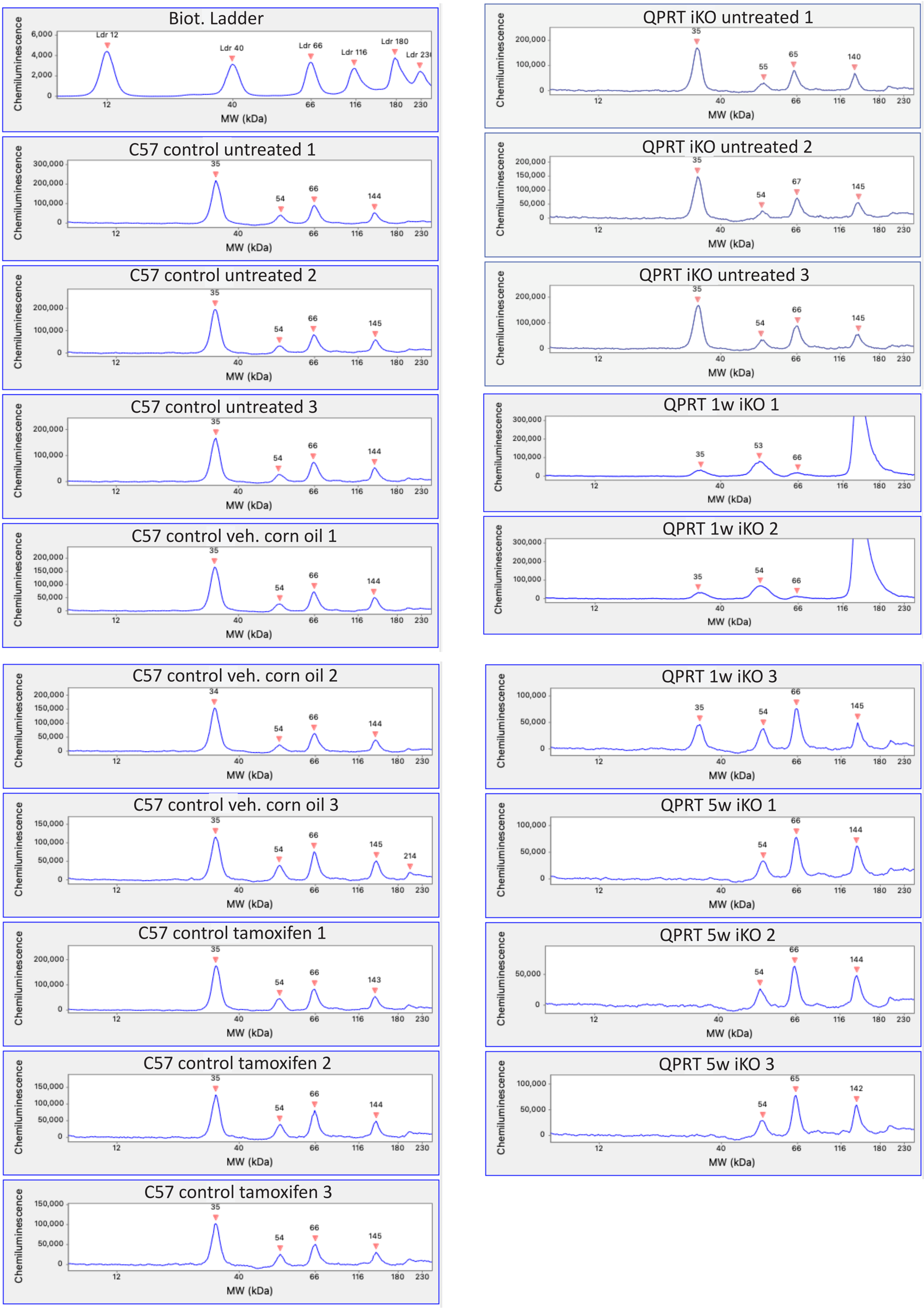

Supplement: S1 Fig — Computer-generated blot images produced by the ProteinSimple WES automated capillary. electrophoresis system. These images are not traditional Western blots. Instead, they are software-generated representations created from the underlying electropherogram peak data to provide a format familiar to readers. The raw electropherogram traces were the actual data analyzed in this study (see Supporting Information). The images shown here are uncropped, unadjusted, and presented exactly as exported from the WES software. (PDF) [file pone.0335906.s003.pdf]
