# Supplementary material for: Renal and hepatic function is preserved following inducible knockout of kynurenine pathway enzymes KMO or QPRT in adult mice
Source: PLoS One. 2025 Dec 4;20(12):e0335906. doi: 10.1371/journal.pone.0335906 (PMC12677463; doi:10.1371/journal.pone.0335906)
Supplement: Attachment — Submitted filename: Response to Reviewers.pdf [file pone.0335906.s005.pdf]

Rebuttal to Reviewer Comments

We thank the reviewers for their constructive and insightful feedback, which has helped us to improve the clarity and rigor of our manuscript. Below, we provide detailed responses to each comment. All corresponding changes have been incorporated into the revised manuscript, with additions or modifications clearly marked.

Reviewer 1:

Comment 1: *Normally, in Introduction section, we try to introduce the research and state the importance of conducting it. We show discrepancies and gaps in previous studies and that we are going to fill the gaps. As readers, we usually don't expect to know about results of the upcoming research. It is recommended to delete this part of introduction.*

Response: We agree with the reviewer and have removed this section from the manuscript.

Comment 2: *In case of control and transgenic mice, although it has been stated in figure caption, it is recommended to state the following issues in methods section as well. 1) How many mice were allocated to each group (Control, KMO, and QPRT)? 2) How many mice were male and how many mice were female?*

Response: We have updated the methods section of the manuscript to address these points by a) emphasising that these details are found in figure legends, and b) including details on number and sex of mix in the appropriate method sections. Changes included in each methods sections detailed below:

- **Transgenic mice**: "Exact number of mice utilised in each experiment is detailed in subsequent methods sections and in figure legends"
- **Blood collection, processing, and profiling**: "Hematology and analytical chemistry profiling was conducted on the same cohort of mice, consisting of a minimum of 9 mice per genotype and treatment, with equal representation of males and females.". "To isolate blood plasma for profiling of blood metabolites via mass spectrometry, blood was drawn from an independent cohort of 6 mice per genotype and treatment, with attempts made to even match the number of males and females"
- **Tissue collection**: "To collect tissue for immunohistochemical and histological analysis, a cohort of mice (3 females and 3 males per genotype and treatment) were perfused with chilled PBS…". "To collect tissue collected for western blot analysis, a second cohort of mice (3 females and 3 males per genotype and treatment) were perfused with chilled PBS only….".

Comment 3: *BUN is not a protein.*

Response: We understand BUN is not a protein and have altered the manuscript text accordingly to correct this error.

Comment 4: *could we think that we had decrease in globulins mainly gamma globulins due to leukopenia induced in transgenic mice while compensated by dehydration and didn't show any change?*

Response: We thank the reviewer for the interesting suggestion on γ-globulins, which raised a possibility we had not previously considered – that a reduction in γ-globulins secondary to leukopenia could be masked by dehydration-related hemoconcentration. To explore this, we examined serum globulin concentrations and calculated albumin-to-globulin (A/G) ratios for each group detailed in Table 1. Globulin levels were unchanged in both KMO-iKO (13.11 ± 2.57 g/L) and QPRT-iKO (12.40 ± 1.90 g/L) mice compared with controls (12.22 ± 1.72 g/L), and A/G ratios remained within the control range (Control = 2.87, KMO-iKO = 2.61, QPRT-iKO = 3.04). Although KMO-iKO mice exhibited a lower total WBC count, lymphocyte counts were not significantly reduced, suggesting that antibody-producing B cells were preserved. Additionally, hematocrit, hemoglobin, and sodium were unchanged in both knockout groups, providing no evidence for hemoconcentration from dehydration. We have added these results to the manuscript discussion to address this interesting hypothesis:

- **Results section**: "To assess if altered hydration status may mask a reduction in globulin, we examined serum globulin concentrations alongside calculated albumin-to-globulin (A/G) ratios. Both globulin and A/G ratio were similar across groups (Control: 12.22 ± 1.72 g/L, A/G = 2.87; KMO-iKO: 13.11 ± 2.57 g/L, A/G = 2.61; QPRT-iKO: 12.40 ± 1.90 g/L, A/G = 3.04), and together with stable hematocrit, hemoglobin, and sodium, this analysis suggested globulin levels were unchanged."
- **Discussion**: "While dehydration can increase the concentration of plasma proteins through hemoconcentration and mask reductions in globulins, mainly γ-globulins, our findings do not support this mechanism. Globulin levels and albumin-to-globulin ratios were unchanged, and markers of hydration status, including hematocrit, hemoglobin, and sodium, were stable. γ-globulins are primarily produced by B lymphocyte derived plasma cells, and as our lymphocyte counts were preserved in both knockout models, therefore a substantial loss of γ-globulin production is unlikely"

Reviewer 2:

Comment 1: *On which bases was the Tamoxifen dose selected for the experiment?*

Response: We selected our tamoxifen dose based on established protocols in our laboratory and in published studies using the CreERT2 system for gene deletion. The chosen regimen represents the highest dose that we and others have determined does not produce observable adverse effects in mice, while reliably achieving maximal recombination efficiency. This was particularly important in the present study, as our goal was to knock out enzymes expressed systemically, across multiple organs, tissues, and cell types. Maximizing recombination efficiency ensured that any phenotypic effects would reflect enzyme loss rather than incomplete recombination. This has been included in our Tamoxifen administration methods section including supporting references:

- "This regimen was selected based on published studies reporting high levels of recombination while being deemed a tolerable dose in adult mice, and a tolerable dose in our mice that did not have a noticeable adverse effects on mouse health or weight"

Comment 2: *For Blood collection, processing, and profiling, the authors didn't mention if blood collected for analytical chemistry was analysed immediately or stored until analysed (and if stored at what temperature and for what duration).*

Response: Blood was analysed immediately after collection. This information has been included in the "Blood collection, processing, and profiling" methods section.

Comment 3: F*or Tissue Collection the authors didn't mention if tissue samples were collected from all groups of mice or from representative sample of each group of mice, for each procedure.*

Response: As per reviewer 1 question 2, we have updated the methods sections to include mouse numbers and sex for each procedure / experiment.

Comment 4: *The reviewer commented on repetition in the description of tamoxifen administration in the methods section.*

Responses: The text has been adjusted:

- Original: "In the following results and discussion, treatments/experimental timepoints are represented relative to the day of tamoxifen administration. For example, tissue collected 1-week post tamoxifen indicates tissue was collected 7-days after the first dose of tamoxifen was administered."
- Adjusted text: "In the following results and discussion, treatments/experimental timepoints are represented relative to the first day of tamoxifen administration."
